# The SENSE trial: etravirine shows lower prevalence and severity of neuropsychiatric adverse events compared to efavirenz in treatment-naïve patients

**DOI:** 10.1186/1758-2652-13-S4-O47

**Published:** 2010-11-08

**Authors:** HJ Stellbrink, S Rugina, C Zagler, S Esser, A Castagna, B Gazzard, A Hill, Y van Delft, S Marks

**Affiliations:** 1 Infektionsmedizinisches Centrum, Hamburg, Germany; 2 Spitalul Clinic de Boli Infectioase, Constanta, Romania; 3 SMZ Baumgartner, Vienna, Austria; 4 Universitätsklinikum, Essen, Germany; 5 Fondazione San Raffaele, Milan, Italy; 6 St Stephens Centre, Chelsea and Westminster Hospital, London, UK; 7 Liverpool University and Tibotec BVBA, Liverpool, UK; 8 Janssen-Cilag, Tilburg, Netherlands

## Background

Efavirenz (EFV) treatment is associated with a range of neuropsychiatric (NPS) adverse events (AEs), which differ in duration and severity.

## Methods

In this double-blind placebo-controlled trial, 157 treatment-naïve patients with HIV RNA >5000 copies/mL, were randomised 1:1 to either etravirine (ETR) 400mg once daily (n=79), or EFV 600mg once daily (n=78), plus two NRTIs. After 12 weeks of randomised treatment, the type and frequency of NPS AEs was compared between treatment arms.

## Results

Overall, the patients were 81% male, 85% Caucasian, with a median age of 36 years. Median baseline CD4 Count was 302 cells/uL, median HIV RNA 4.8 log10 copies/mL. In the primary analysis, 13/79 patients (16.5%) in the ETR arm, versus 36/78 (46.2%) in the EFV arm, showed at least one Grade 1-4 treatment-emergent drug-related NPS AE (p<0.001). The most common nervous system was dizziness, reported for 3 patients in the ETR arm versus 15 in the EFV arm. The most common psychiatric adverse events were sleep disorders, reported in 7 patients in the ETR arm versus 25 patients in the EFV arm. The prevalence of Grade 1-4 all cause NPS AEs showed a peak at Week 2 (21.5% in the ETR arm and 43.6% in the EFV arm), but at the Week 12 visit, the percentage with an ongoing Grade 1-4 all cause NPS AE remained different between the arms (21.7% with ETR and 35.7% with EFV). In the ETR arm, 29 all cause NPS adverse events were reported: 20 Grade 1, 7 Grade 2 and 2 Grade 3. In the EFV arm, 93 NPS adverse events were reported: 55 Grade 1, 34 Grade 2 and four Grade 3. New medication for NPS adverse events was started for 7.6% of patients in the ETR arm versus 16.7% of patients in the EFV arm. One patient in the ETR arm and five in the EFV arm discontinued randomized treatment with NPS AE’s.

## Conclusions

In the SENSE trial, first-line treatment with ETR 400mg once daily +2NRTIs led to significantly fewer NPS AEs, compared with EFV + 2NRTIs. These NPS AEs were mainly Grade 1 or 2 in severity. The difference between the arms emerged at Week 2, but persisted through Week 12.

